# Supplementary material for: The Effects of Nitrogen Fertilizer on the Aroma of Fresh Tea Leaves from Camellia sinensis cv. Jin Xuan in Summer and Autumn
Source: Foods. 2024 Jun 5;13(11):1776. doi: 10.3390/foods13111776 (PMC11172281; doi:10.3390/foods13111776)
Supplement: Supplementary file 1 [file foods-13-01776-s001.zip › foods-3023472-supplementary.pdf]

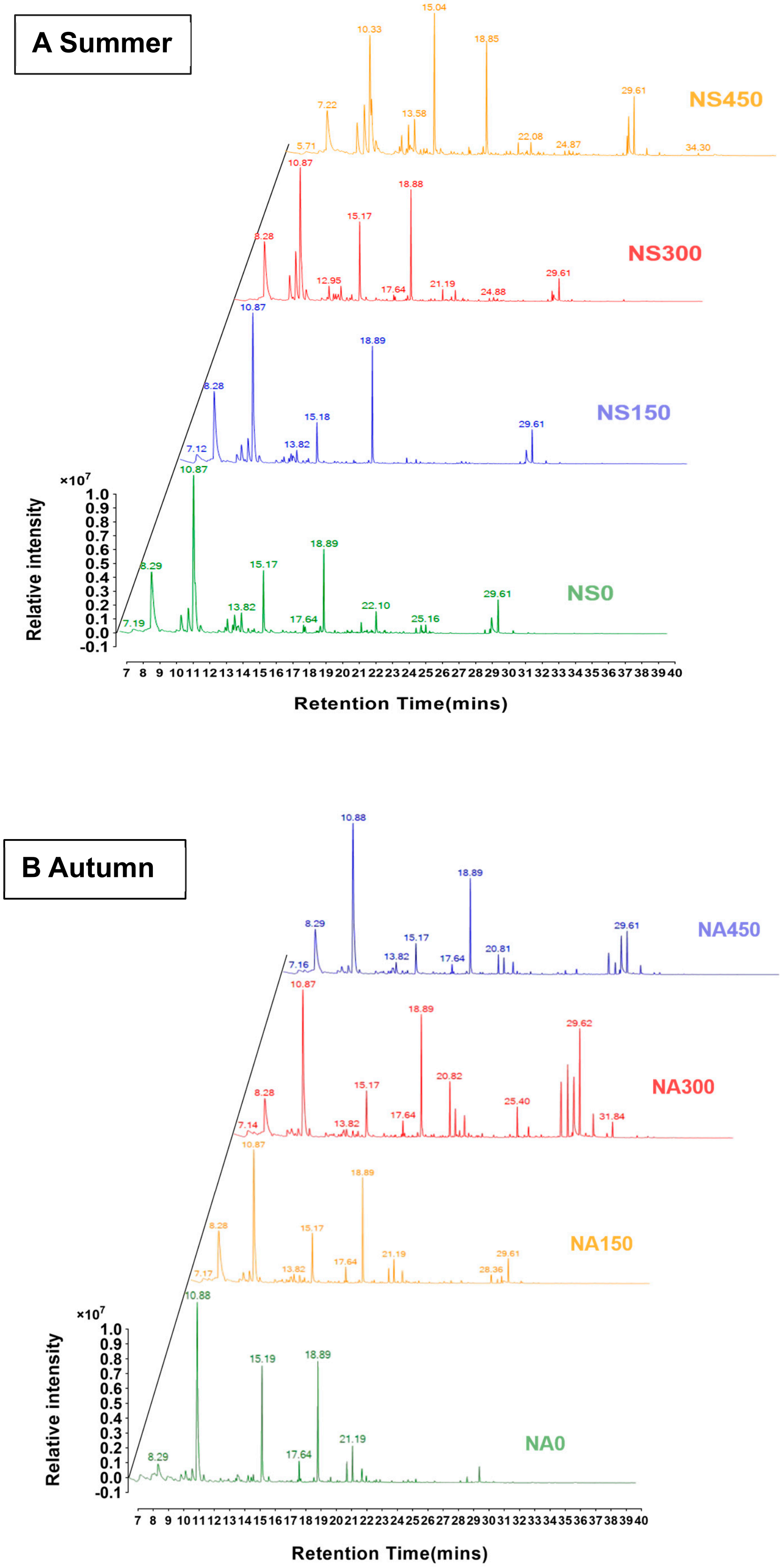

Figure S1. The GC-MS base peak chromatogram of volatile substances. The relative intensity is converted using the peak area normalization method.

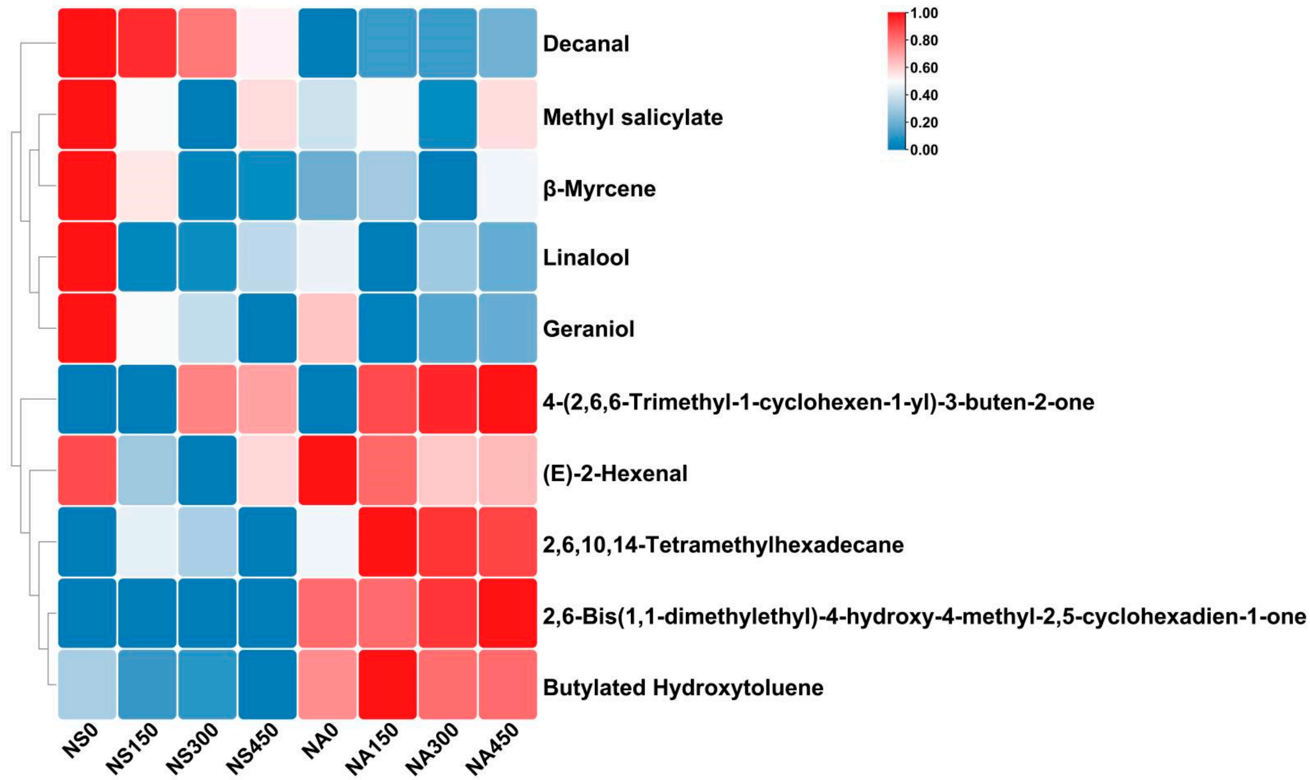

Figure S2. Heat map of the top 10 volatile organic compounds in fresh Jin Xuan leaves.

Table S1. The content of identified volatile compounds.

Compound name	Aroma description	CAS	RI[a]/RI[b]	Identification[c]	Mean content ± Standard deviation							
					NS0	NS150	NS300	NS450	NA0	NA150	NA300	NA450
**Alcohols**												
Linalool	floral, sweet	000078-70-6	1102/1104	MS, RI	963.54±281.71	405.40±56.64	416.42±169.01	541.30±62.17	591.05±95.62	391.28±337.03	510.89±56.03	461.74±401.83
(E)-2,6-Dimethylocta-3,7-diene-2,6-diol	woody	051276-34-7	1191/1191	MS, RI	72.96±9.49	44.23±7.58	0.00±0.00	0.00±0.00	0.00±0.00	0.00±0.00	0.00±0.00	0.00±0.00
(Z)-2,6-Octadien-1-ol, 3,7-dimethyl-	fresh, citrus, floral, green, lemon-like	000106-25-2	1229/1229	MS, RI	16.70±8.94	11.12±2.91	6.87±2.88	0.00±0.00	9.40±3.81	39.04±53.82	6.65±1.87	9.02±2.82
Geraniol	Rose-like, sweet, honey-like	000106-24-1	1256/1267	MS, RI	277.19±171.13	154.02±91.15	134.66±49.60	87.36±14.04	181.10±76.97	88.99±70.15	105.25±29.77	108.36±13.61
τ-Muurolol		019912-62-0	1646/1640	MS, RI	25.56±2.86	7.91±1.03	9.35±2.14	6.86±0.50	8.59±2.95	0.00±0.00	0.00±0.00	0.00±0.00
α-Cadinol	woody, herb-like, flowery	000481-34-5	1659/1650	MS, RI	19.70±2.23	4.53±1.25	5.29±1.23	4.80±0.17	3.37±0.65	7.22±9.78	1.41±0.30	2.45±0.63
(3R,6S)-2,2,6-Trimethyl-6-vinyltetrahydro-2H-pyran-3-ol	woody	039028-58-5	1175/1183	MS, RI	0.00±0.00	42.20±7.17	29.67±15.36	22.64±4.20	13.91±1.89	0.00±0.00	0.00±0.00	0.00±0.00
Cedrol	Woody	00007	1607/1607	MS, RI	0.00±0.0	1.34±0.1	0.00±0.0	0.00±0.0	1.20±0.3	0.00±0.0	1.60±0.7	0.00±0.0

		7-53-2			0	7	0	0	8	0	8	0
α,2,6,6-Tetramethyl-1-cyclohexene-1-propanol	woody,flowery，camphoric	003293-47-8	1448/1449	MS, RI	0.00±0.00	0.00±0.00	0.00±0.00	0.00±0.00	3.00±0.85	2.30±0.28	2.46±0.78	2.45±0.26
(E)-1,6,10-Dodecatrien-3-ol, 3,7,11-trimethyl-	floral, green, citrus, woody, waxy	040716-66-3	1565/1565	MS, RI	0.00±0.00	0.00±0.00	0.00±0.00	0.00±0.00	8.44±4.63	0.00±0.00	0.00±0.00	7.96±1.70
**Aldehydes**												
(E)-2-Hexenal	green, fresh, fruity	006728-26-3	862/855	MS, RI	1741.48±474.66	1153.67±437.81	945.79±126.67	1416.79±87.87	1885.15±1031.74	1669.86±376.03	1450.49±288.94	1481.29±459.85
4-Ethylbenzaldehyde	bitter-like, sweet	004748-78-1	1165/1206	MS, RI	6.12±0.24	3.96±1.54	0.00±0.00	0.00±0.00	6.74±0.44	0.00±0.00	0.00±0.00	0.00±0.00
Decanal	citrus, fatty, green	000112-31-2	1206/1200	MS, RI	79.97±18.02	73.64±20.11	57.24±23.01	36.92±9.79	15.67±0.36	18.98±12.24	18.94±5.14	22.02±8.66
3-Ethylbenzaldehyde	fruity, sweet, cherry	034246-54-3	1165/1168	MS, RI	0.00±0.00	0.00±0.00	0.00±0.00	2.81±0.46	0.00±0.00	0.00±0.00	8.29±1.77	10.16±0.48
2,6,6-Trimethyl-1-cyclohexene-1-carboxaldehyde	herbal, clean, rose-like, fruity	000432-25-7	1222/1214	MS, RI	0.00±0.00	0.00±0.00	11.50±3.40	0.00±0.00	17.28±3.54	0.00±0.00	15.49±3.15	9.39±2.70
**Ketones**												
Precocene I		017598-02-6	1465/1471	MS, RI	9.88±0.75	5.87±0.83	4.60±1.57	3.29±1.34	0.00±0.00	0.00±0.00	0.00±0.00	0.00±0.00

trans-β-Ionone	violet	000079-77-6	1488/1490	MS, RI	28.38±1.16	13.80±2.98	0.00±0.00	0.00±0.00	37.72±5.10	0.00±0.00	0.00±0.00	0.00±0.00
2-Pentadecanone, 6,10,14-trimethyl-	jasmine-like, oily, herbal	000502-69-2	1839/1847	MS, RI	17.86±12.71	23.57±3.81	14.63±3.92	7.22±3.76	7.14±8.50	9.51±4.00	8.85±3.84	9.88±3.34
4-(2,6,6-Trimethyl-1-cyclohexen-1-yl)-3-buten-2-one	floral, violet, fruity,woody	014901-07-6	1488/1488	MS, RI	0.00±0.00	0.00±0.00	19.57±2.80	14.77±3.63	0.00±0.00	31.86±1.92	42.76±6.85	50.31±15.74
(E)-5,9-Undecadien-2-one, 6,10-dimethyl-	magnolia, green	003796-70-1	1454/1458	MS, RI	0.00±0.00	0.00±0.00	0.00±0.00	2.59±0.09	0.00±0.00	3.67±0.25	0.00±0.00	0.00±0.00
(R)-5,6,7,7a-Tetrahydro-4,4,7a-trimethyl-2(4H)-benzofuranone	musky or coumarin-like	017092-92-1	1534/1525	MS, RI	0.00±0.00	0.00±0.00	0.00±0.00	3.90±0.41	4.54±0.63	0.00±0.00	0.00±0.00	0.00±0.00
7,9-Di-tert-butyl-1-oxaspiro(4,5)deca-6,9-diene-2,8-dione		082304-66-3	1921/1929	MS, RI	0.00±0.00	0.00±0.00	0.00±0.00	1.62±0.05	1.47±0.27	3.89±2.91	3.75±2.55	2.52±0.04
α-Ionone	floral, violet-like, powdery, berry-like	000127-41-3	1430/1427	MS, RI	0.00±0.00	0.00±0.00	0.00±0.00	0.00±0.00	7.69±2.34	3.22±0.29	6.05±1.50	7.21±3.47

2,6-Bis(1,1-dimethylethyl)-2,5-cyclohexadiene-1,4-dione		000719-22-2	1469/1472	MS, RI	0.00±0.00	0.00±0.00	0.00±0.00	0.00±0.00	2.94±0.50	2.00±0.70	2.44±0.75	2.92±0.68
2,6-Bis(1,1-dimethylethyl)-4-hydroxy-4-methyl-2,5-cyclohexadien-1-one		1000401-12-0	1473/1470	MS, RI	0.00±0.00	0.00±0.00	0.00±0.00	0.00±0.00	37.68±8.47	37.75±7.90	61.53±26.36	85.15±45.33
**Hydrocarbon**												
2,6,10-Trimethyltridecane		003891-99-4	1461/1461	MS, RI	2.80±0.30	0.00±0.00	0.00±0.00	0.00±0.00	0.00±0.00	1.84±0.04	1.99±0.26	1.61±0.31
2,6,10,14-Tetramethylpentadecane		001921-70-6	1704/1704	MS, RI	0.00±0.00	0.00±0.00	0.00±0.00	0.00±0.00	9.01±5.71	22.52±25.89	13.55±6.12	15.34±1.13
2,6,10,14-Tetramethylhexadecane		000638-36-8	1798/1811	MS, RI	0.00±0.00	6.51±5.97	3.20±2.44	0.00±0.00	7.48±1.91	88.08±104.36	64.75±40.50	55.41±38.63
**Alkene**												
β-Myrcene	sweet orange-like	000123-35-3	938/955	MS, RI	34.09±13.28	22.84±4.42	14.27±2.24	14.59±3.90	16.41±8.65	18.20±6.25	13.81±5.30	21.26±3.81
β-Ocimene	sweet floral	013877-91-3	1048/1027	MS, RI	15.83±1.00	0.00±0.00	0.00±0.00	0.00±0.00	0.00±0.00	0.00±0.00	7.42±1.10	0.00±0.00
α-Cubebene	herbal waxy	017699-14-8	1352/1354	MS, RI	18.29±3.17	0.00±0.00	9.32±2.76	0.00±0.00	0.00±0.00	0.00±0.00	0.00±0.00	0.00±0.00
Caryophyllene	woody, green, spicy, terpenic	000087-44-5	1423/1417	MS, RI	9.36±0.71	4.15±1.03	0.00±0.00	4.11±1.42	0.00±0.00	0.00±0.00	0.00±0.00	0.00±0.00

γ-Muurolene	woody	030021-74-0	1480/1474	MS, RI	3.71±0.91	0.00±0.00	0.00±0.00	0.00±0.00	1.48±0.06	0.00±0.00	0.00±0.00	0.00±0.00
Bicyclosesquiphellandrene	spicy, pungent, pepper-like	054324-03-7	1496/1498	MS, RI	7.30±1.00	3.45±0.41	3.50±0.94	0.00±0.00	0.00±0.00	0.00±0.00	0.00±0.00	0.00±0.00
α-Muurolene	woody	031983-22-9	1503/1517	MS, RI	7.77±1.48	2.26±0.42	2.94±0.83	0.00±0.00	2.02±0.52	0.00±0.00	0.00±0.00	0.00±0.00
cis-Calamenene		072937-55-4	1527/1531	MS, RI	58.31±4.45	18.74±2.76	23.49±5.56	6.65±4.49	14.51±1.80	9.40±1.21	8.17±1.79	0.00±0.00
α-Calacorene	woody	021391-99-1	1547/1542	MS, RI	5.39±0.52	2.09±0.35	2.17±0.45	1.64±0.73	2.15±0.41	1.58±0.23	0.00±0.00	2.06±0.75
(Z)-3,7-Dimethyl-1,3,6-octatriene		003338-55-4	1049/1039	MS, RI	0.00±0.00	11.98±0.97	0.00±0.00	0.00±0.00	5.61±4.33	0.00±0.00	0.00±0.00	22.17±23.06
(E,Z)-2,6-Dimethyl-2,4,6-octatriene		007216-56-0	1131/1131	MS, RI	0.00±0.00	0.00±0.00	0.00±0.00	0.00±0.00	6.97±1.52	8.99±6.30	3.41±1.04	0.00±0.00
**Heterocyclic**												
[(3as,3bR,4bS,7S,7aS*)]-7-Methyl-4-(propan-2-yl)-octahydro-3-methylene-1H-cyclopenta[1,3]cyclopropa[1,2]benzene		013744-15-5	1392/1388	MS, RI	4.39±0.66	1.77±0.41	2.68±1.03	0.00±0.00	0.00±0.00	0.00±0.00	0.00±0.00	0.00±0.00
1-Isopropyl-4,7-dimethyldecahydronaphthalene		016729-00-3	1477/1481	MS, RI	2.46±0.56	0.00±0.00	1.82±0.08	0.00±0.00	0.00±0.00	0.00±0.00	0.00±0.00	0.00±0.00

**Esters**												
Methyl salicylate	peppermint, minty, fresh, sweet	000119-36-8	1197/1187	MS, RI	61.87±47.52	23.48±8.26	8.70±2.60	27.39±10.59	19.11±6.62	23.95±14.46	9.81±3.58	27.14±13.37
Dibutyl phthalate		000084-74-2	1964/1967	MS, RI	4.22±0.53	6.07±0.63	3.62±1.04	5.42±1.79	0.00±0.00	4.64±4.03	5.69±2.47	9.32±5.60
2,2,4-Trimethyl-1,3-pentanediol diisobutyrate		006846-50-0	1599/1587	MS, RI	0.00±0.00	7.30±1.91	0.00±0.00	7.69±0.83	4.50±0.40	0.00±0.00	0.00±0.00	0.00±0.00
(Z)-3-Hexenyl hexanoate	green, waxy,winey, grassy	031501-11-8	1382/1380	MS, RI	6.97±0.61	0.00±0.00	5.50±0.79	0.00±0.00	0.00±0.00	0.00±0.00	0.00±0.00	0.00±0.00
**Others**												
Benzyl nitrile	bitter almond like	000140-29-4	1144/1143	MS, RI	12.99±4.85	0.00±0.00	6.39±4.40	0.00±0.00	0.00±0.00	0.00±0.00	0.00±0.00	0.00±0.00
Butylated Hydroxytoluene		000128-37-0	1514/1517	MS, RI	11.93±1.02	7.89±2.85	7.79±2.77	6.39±1.72	27.02±4.64	43.17±20.40	30.55±10.24	30.92±11.52
1,6-Dimethyl-4-isopropyl-naphthalene		000483-78-3	1679/1674	MS, RI	7.40±0.78	5.26±0.49	4.32±0.84	3.83±0.13	0.00±0.00	2.05±0.30	0.00±0.00	1.11±0.85
2,6-Di-tert-butyl-4-(1-oxopropyl)phenol		014035-34-8	1639/1635	MS, RI	0.00±0.00	1.50±0.25	1.56±0.15	1.61±0.08	1.62±0.38	0.00±0.00	0.00±0.00	1.60±0.24

[a] Retention index of compounds on HP-5MS.

[b] Retention index of compounds in reference.

[c] "MS" mass spetrum comparison using NIST17 library. "RI" retention index in agreement with literature value. "STD" confirmed by authenic standards.
